# Mice, myeloid cells, and dengue: a new model for unraveling vascular leakage mysteries

**DOI:** 10.3389/fmicb.2024.1367672

**Published:** 2024-03-14

**Authors:** Takeshi Kurosu, Yusuke Sakai, Yasusi Ami, Masayuki Shimojima, Tomoki Yoshikawa, Shuetsu Fukushi, Noriyo Nagata, Tadaki Suzuki, Hideki Ebihara, Masayuki Saijo

**Affiliations:** ^1^Department of Virology I, National Institute of Infectious Diseases, Tokyo, Japan; ^2^Department of Pathology, National Institute of Infectious Diseases, Tokyo, Japan; ^3^Management Department of Biosafety, Laboratory Animal, and Pathogen Bank, National Institute of Infectious Diseases, Tokyo, Japan

**Keywords:** severe dengue, myeloid cells, vascular leakage, cytokine storm, bone marrow suppression, pathogenic mechanisms, viral adaptation

## Abstract

**Introduction:**

Severe dengue is thought to be caused by an excessive host immune response.

**Methods:**

To study the pathogenesis of severe dengue, we developed a novel model using LysM Cre^+^*Ifnar*^flox/flox^ mice carrying depleted *Ifnar* expression only in subsets of murine myeloid cells.

**Results:**

Although dengue virus (DENV) clinical isolates were not virulent in LysM Cre^+^*Ifnar*^flox/flox^ mice, mouse-adapted DV1-5P7Sp and DV3P12/08P4Bm, which were obtained by passaging the spleen or bone marrow of mice, demonstrated 100% lethality with severe vascular leakage in the liver and small intestine. DV1-5P7Sp and DV3P12/08P4Bm harbored five and seven amino acid substitutions, respectively. Infection also induced neutrophil infiltration in the small intestine, and increased expression of IL-6 and MMP-8 and blockade of TNF-α signaling protected the mice, as demonstrated in a previous severe dengue mouse model using C57/BL6 mice lacking both IFN-α/β and IFN-γ receptors. Notably, the new models with DV1-5P7Sp and DV3P12/08P4Bm showed an increased proliferative capacity of the adapted viruses in the thymus and bone marrow.

**Discussion:**

These observations suggest that myeloid cell infection is sufficient to trigger cytokine storm-induced vascular leakage. This model can refine the factors involved in the pathology of severe dengue leading to vascular leakage.

## 1 Introduction

Dengue is caused by four serotypes, dengue virus (DENV) 1–4, and occasionally causes severe disease in humans. Typical symptoms of severe dengue include severe vascular leakage leading to pleural or peritoneal effusion, severe bleeding, or severe organ impairment^1^ accompanied by significant thrombocytopenia (Guzman et al., 2007). The defining feature of severe dengue is characterized by the disruption of the vascular barrier function, leading to vascular leakage (Yacoub and Wills, 2014); however, the pathophysiological mechanism is not fully understood. To understand this mechanism, it is necessary to use an animal model, because severe dengue is believed to be caused by the host immune response. However, developing mouse models to study dengue pathogenesis has been challenging because of the inefficient replication of dengue viruses in mice and their lack of virulence. Shresta et al. (2006) reported a model using the adapted DENV-2 D2S10 in 129/Sv mice doubly deficient in interferon-alpha/beta (IFN-α/β) and IFN-γ receptors (AG129). Sarathy et al. (2015) reported a model using the DENV-3 C0360/94 strain in AG129 mice. Furthermore, we reported another model using the DENV-3 P12/08 (DV3P12/08) clinical isolate in C57/BL6 mice lacking IFN-α/β and IFN-γ receptors (IFN-α/β/γR KO mice) (Phanthanawiboon et al., 2016; Kurosu et al., 2023b), as well as a model with a chimeric flavivirus (Kurosu et al., 2020, 2023a). Zellweger et al. (2010) also developed a model using the DENV-2S221 strain in A129 mice. In addition, humanized mice that received human CD34^+^ hematopoietic progenitor cells have been developed (Mota and Rico-Hesse, 2009; Sridharan et al., 2013; Frias-Staheli et al., 2014). However, the limitations of these study have been discussed, as the mice used therein were considered to have weak systemic immunity.

Pinto et al. (2015) developed another mouse model using LysM Cre^+^*Ifnar*^flox/flox^ mice carrying depleted *Ifnar* expression only in subsets of murine myeloid cells. LysM Cre^+^*Ifnar*^flox/flox^ mice infected with DENV-2 D2S20 showed partial lethality and characteristic human symptoms such as vascular leakage, hypercytokinemia, liver damage, and thrombocytopenia under antibody-dependent enhancement (ADE) conditions. However, LysM Cre^+^*Ifnar*^flox/flox^ mice did not show lethality or other dengue-like symptoms when administered the virus alone. Here, we present a further developed LysM Cre^+^*Ifnar*^flox/flox^ mouse model using DV1-5P7Sp or DV3P12/08P4Bm dengue viruses that were specifically passaged in mouse organs. DV1-5P7Sp and DV3P12/08P4Bm were produced by passage of parental DENV-1 DV1-5 and DV3P12/08 clinical isolates into the spleen and bone marrow, respectively. DV1-5P7Sp and DV3P12/08P4Bm demonstrated 100% lethality with dramatic gross changes in the liver and small intestine, vascular leakage, and bone marrow suppression. DV1-5P7Sp and DV3P12/08P4Bm increased viral production in the thymus and bone marrow of LysM Cre^+^*Ifnar*^flox/flox^ mice compared to parental DV1-5 and DV3P12/08 mice, respectively. Five and seven amino acid substitutions were found in DV1-5P7Sp and DV3P12/08P4Bm, respectively. Blockade of TNF-α signaling protected LysM Cre^+^*Ifnar*^flox/flox^ mice from lethal infections. LysM Cre^+^*Ifnar*^flox/flox^ mice infected with DV1-5P7Sp or DV3P12/08P4Bm showed infiltration of large numbers of neutrophils into the small intestine and high levels of IL-6, MMP-8, and MMP-3.

## 2 Materials and methods

### 2.1 Ethics statement

All experiments involving animals were performed in animal biological safety level 2 containment laboratories at the National Institute of Infectious Diseases (NIID) in Japan in accordance with the animal experimentation guidelines of the NIID. The protocols were approved by the Institutional Animal Care and Use Committee of the NIID (Nos. 115,064, 118,009, 121,003, and 121,005). Trained laboratory personnel anesthetized the mice via intraperitoneal injection of a mixture of medetomidine, midazolam, and butorphanol prior to viral injection.

### 2.2 Virus and cells

**Figure 1:**
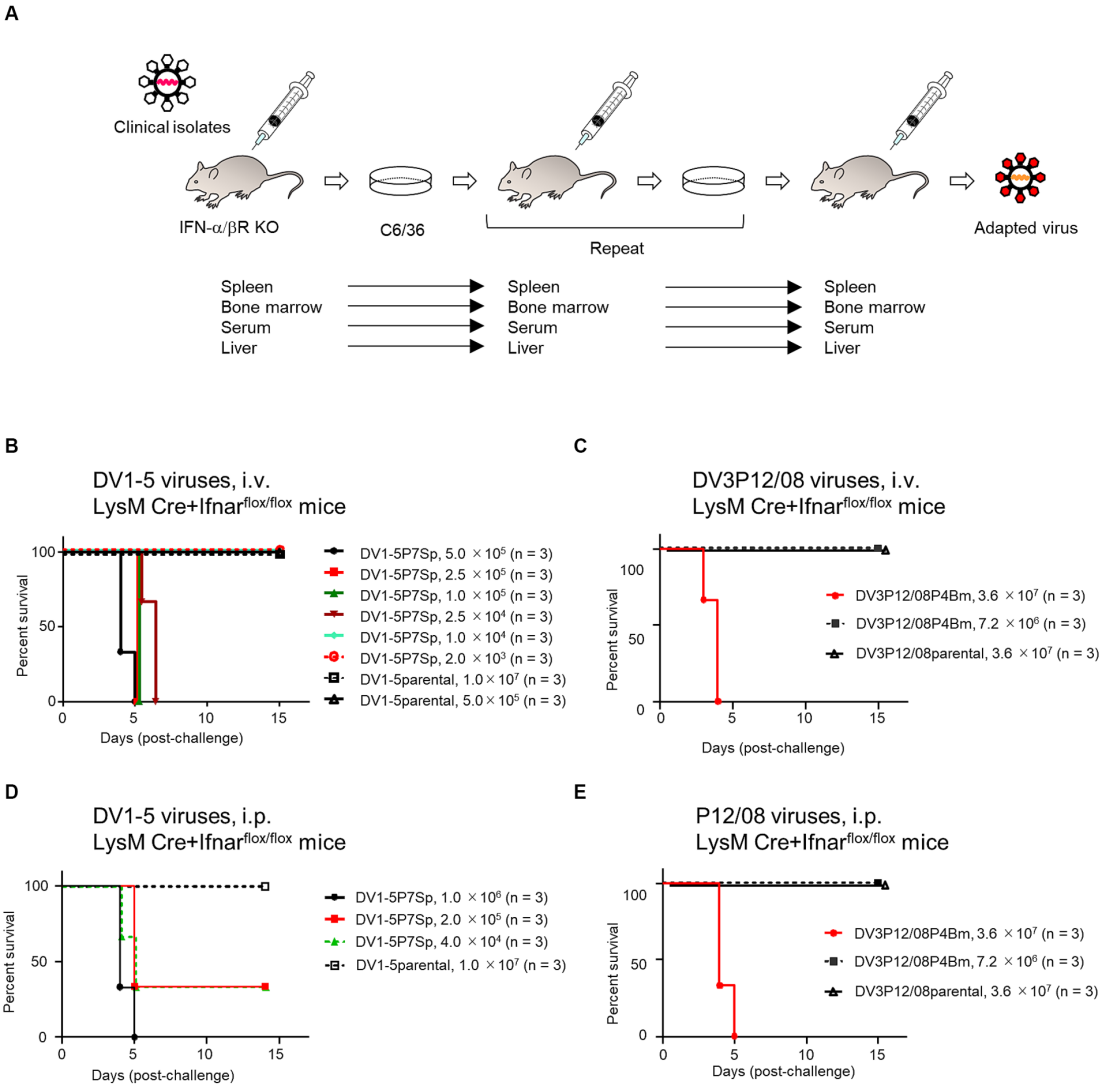
Survival rates of LysM Cre + Ifnar^flox/flox^ mice infected with various DENVs. **(A)** Virus adaptation. IFN-α/βR KO mice were intravenously inoculated with DV1-5 or DV3P12/08. The spleen, liver, bone marrow, or serum was collected 3–5 days post-infection. Organs were homogenized in PBS and inoculated in C6/36 cells. Sera were diluted in PBS and inoculated in C6/36 cells. IFN-α/βR KO mice were intravenously inoculated with the culture supernatants. DV1-5P7Sp or DV3P12/08P4Bm was obtained by repeating this procedure seven or four times in the spleen or bone marrow, respectively. The supernatants from C6/36 cells were used to examine virulence in LysM Cre + Ifnar^flox/flox^ mice. **(B)** Groups of LysM Cre + Ifnar^flox/flox^ mice (10–12 weeks old) were intravenously infected with DV1-5P7Sp at doses ranging from 2.0 × 10^3^ to 5.0 × 10^5^ focus-forming units per mouse (FFU/mouse) or DV1-5 at does ranging from 5.0 × 10^5^ to 1.0 × 10^7^, and their survival was monitored. **(C)** Groups of LysM Cre + Ifnar^flox/flox^ mice (10–12 weeks old) were intravenously infected with DV3P12/08P4Bm at doses 7.2 × 10^6^ or 3.6 × 10^7^ FFU/mouse, and their survival was monitored. **(D)** Groups of LysM Cre + Ifnar^flox/flox^ mice (10–12 weeks old) were intraperitoneally infected with DV1-5P7Sp at doses ranging from 4.0 × 10^4^ to 1.0 × 10^6^ FFU/mouse, and their survival was monitored. **(E)** Groups of LysM Cre + Ifnar^flox/flox^ mice (10–12 weeks old) were intraperitoneally infected with DV3P12/08P4Bm at doses 7.2 × 10^6^ or 3.6 × 10^7^ FFU/mouse, and their survival was monitored. Kaplan–Meier survival curves show the percentage of mice surviving at the specified days post-infection.

The parental virus strains DV1-5 and DV3P12/08, which were derived from patients infected with DENV-1 or DENV-3 in Thailand (Pambudi et al., 2013), were propagated in mosquito C6/36 cells. For adaptation of DENV to mice, DV1-5 or DV3P12/08 was intravenously infected in IFN-α/βR KO mice (Figure 1A). Four to five days post infection (p.i.), the viruses were isolated from the spleen, liver, bone marrow, or serum. The C6/36 cells were inoculated with 10% homogenates of each organ in phosphate-buffered saline (PBS), and the culture supernatants were collected on days 5–7 in order to perform further inoculation to IFN-α/βR KO mice. DV1-5P7Sp treatment was repeated seven times in the spleen. DV1-5P3Serum, DV3P12/08P4Bm, and DV3P12/08P4Li were passaged in the serum, bone marrow, or liver three, four, and four times, respectively. Virus stocks were propagated in C6/36 cells, and culture supernatants were kept at −80°C until use. C6/36 cells were cultured in L-15 medium containing 10% fetal calf serum (FCS) and 0.3% BactoTM Tryptose Phosphate Broth (Becton Dickinson, Sparks Glencoe, MD, United States). Vero cells were cultured in Eagle’s minimum essential medium (Nacalai Tesque, Kyoto, Japan) supplemented with 10% FCS.

### 2.3 Mouse experiments

IFN-α/βRKO mice, lacking type I IFN receptors, IFN-α/β/γRKO mice, lacking both type I and type II IFN receptors (Phanthanawiboon et al., 2016), and LysM Cre^+^*Ifnar*^flox/flox^ mice (Pinto et al., 2015), kindly provided by Dr. Shresta, La Jolla Institute for Immunology, were bred and housed in ventilated cages and kept under specific pathogen-free conditions. Male and female mice aged 8 and 12 weeks were used in this study. Mice were anesthetized by intraperitoneal (i.p.) injection of medetomidine, midazolam, and butorphanol tartrate (final concentrations of 0.3 mg/kg, 4 mg/kg, and 5 mg/kg, respectively) and then challenged intraperitoneally or intravenously with appropriate units (FFU) of DENVs. Following inoculation, the mice were weighed and visually monitored at least once daily to score morbidity. Mice were euthanized with isoflurane at the time of sample collection when they became moribund or died because of difficulty eating or drinking.

### 2.4 Focus-forming assay

Focus forming units (FFUs) were used to estimate infectivity. Supernatants collected from the cultures were serially 10-fold diluted in minimum essential medium (MEM) and transferred to Vero cell monolayers in 96-well microplates. After incubation for 2 h at 37°C, the infected cells were further incubated with 2% carboxyl-methylcellulose in 2% fetal bovine serum-MEM for 72 h. The infected cells were washed five times, fixed with 3.7% formaldehyde solution, and permeabilized by treatment with 0.1% Triton X-100 prior to incubation with an anti-E monoclonal antibody (mAb, D23-1G7C2) (Kurosu et al., 2020). After overnight incubation at 4°C, the infected cells were further incubated with peroxidase-conjugated AffiniPure Rabbit anti-human immunoglobulin G (H + L) (309-035-003, Jackson ImmunoResearch, PA, United States) for 45 min. The culture plates were washed with PBS five times. After washing with PBS, the infected cells were visualized by reacting with H_2_O_2_-diaminobenzidine (Sigma-Aldrich, St. Louis, MO, United States), and foci were counted using a light microscope.

### 2.5 Quantitation of vascular permeability

Vascular leakage was examined by intravascular administration of Evans Blue (Sigma-Aldrich) as previously described (Phanthanawiboon et al., 2016). Briefly, Evans Blue (0.2 mL of a 0.5% solution in PBS) was injected intravenously into moribund mice on day 4 p.i. After 2 h, the mice were anesthetized (by i.p. injection of medetomidine, midazolam, and butorphanol tartrate at final concentrations of 0.3 mg/kg, 4 mg/kg, and 5 mg/kg, respectively), euthanized by exsanguination, and perfused extensively with PBS. Liver and small intestine samples were collected. Evans Blue was extracted from the organs by incubation in 1 mL of formamide (Sigma-Aldrich) for 24 h, followed by centrifugation at 3,000 × g for 10 min, and 150 μL of supernatant was collected. The concentration of Evans Blue in each organ was quantified by measuring the absorbance at 620 nm using a Corona Grating Microplate Reader SH-9000 (Corona Electric Co., Ltd.). The results were expressed as optical density per gram of tissue.

### 2.6 Histopathology and immunohistochemistry

Mice were anesthetized and perfused with 10 mL of 10% phosphate-buffered formalin. The liver and small intestine were harvested and fixed. Fixed tissues were embedded in paraffin, sectioned, and stained with hematoxylin and eosin (H&E). For immunostaining, 4 μm thick tissue sections were deparaffinized and heated at 121°C for 15 min in pH6 citrate buffer as antigen retrieval. After washing with phosphate-buffered saline (PBS), endogenous peroxidase was quenched with 3% hydrogen peroxide in PBS. After blocking with 5% skim milk in PBS for 30 min, the tissue sections were incubated at 4°C overnight with rabbit polyclonal anti-CD11b antibody (EPR1344, Abcam) or rabbit anti-cleaved-caspase3 antibody (5E1E, Cell signaling technology). After washing with PBS, sections were incubated with Histofine Simple Stain mouse MAX PO (R) (Nichirei Bioscience). Positive signals were visualized using peroxidase-diaminobenzidine reaction, and the sections were counterstained with hematoxylin. The sections were then scanned using a virtual slide scanner Olympus VS200 and examined using the virtual slide viewer OlyVIA software. Positive cells in the captured images of the tissue section were counted using QuPath software (RRID: SCR_018257). Three photographs were analyzed for each section.

### 2.7 Sequence analysis

DV1-5, DV1-5P7Sp, DV3P12/08, and DV3P12/08P4Bm were obtained from the culture medium of C6/36 cells. Viral RNA (vRNA) was extracted using the High Pure Viral RNA Kit (Roche). Viral cDNA was synthesized using SuperScript III (Invitrogen) and specific primers (Table 1). The viral genome was amplified by PCR using PrimeSTAR MAX DNA polymerase (Takara) with a set of primers (Supplementary Table S2). Nucleotide sequencing analysis was carried out using the indicated primer sets (Supplementary Table S2), the BigDye Terminator v3.1 Cycle Sequencing kit, and a 3500xL Genetic Analyzer (ABI). Data were analyzed using the Sequencher 4.9 software (GeneCodes Corp., Ann Arbor, MI, United States). Multiple sequence alignments were performed using the ClustalW software. The GenBank accession numbers for DV1-5, DV1-5P7Sp, DV3P12/08, and DV3P12/08P4Bm are LC793494, LC793495, LC793496, and LC793497, respectively.

**Table 1:** Challenge of adapted DENV in IFN-α/βR KO mice.

Virus	Titer (FFU)	Number^a^	% Fatality	Period^b^
DV1-5P7Sp	3.6 × 10^7^	3	100	5–6
DV1-5P3Ser	1.3 × 10^6^	3	0	—^c^
DV3P12/08P4Bm	1.3 × 10^6^	3	100	5–6
DV3P12/08P4Li	6.0 × 10^6^	3	100	5–6

a Numbers of mice used for challenge.

b Period indicates the days when mice condition reached the end point.

c All mice survived until days 18 post-infection without symptom.

### 2.8 Quantitative RT-PCR analyses of host genes and DENV RNA

The thymus, liver, spleen, kidney, small intestine, large intestine, brain, bone marrow, and peritoneal exudate cells (PEC) were homogenized using a TissueLyser II (Qiagen). For cDNA synthesis, total RNA was isolated from tissue homogenates using the TRIzol reagent (Thermo Fisher Scientific). One-step real-time quantitative RT-PCR amplification with SYBR Green I was performed using a Light Cycler (Roche) and One Step SYBR PrimeScript RT-PCR Kit II (Takara). The final concentration of each PCR primer was 0.08 μM, and the concentration of total RNA was 8 μg/mL, with a reaction volume of 12.5 μL. The conditions for reverse transcription were as follows: 42°C for 5 min, followed by 95°C for 10 s. PCR amplification used 45 cycles of 95°C for 5 s, 55°C for 30 s, and 72°C for 30 s. The conditions for reverse transcription were 42°C for 5 min, followed by 95°C for 10 s. PCR amplification used 45 cycles of 95°C for 5 s, followed by 60°C for 20 s. To quantify RNA derived from organs, the amounts were normalized to that of total RNA from the corresponding organs from mock-infected mice. Data were analyzed using LightCycler 96 Software ver. 1.1.0.1320 (Roche). Primer sets and probes have been described previously (Kurosu et al., 2023b).

For quantification of viral RNA (vRNA) isolated from the tissue homogenate, total RNA was adjusted to 100 μg/mL for use in real-time PCR. RNA was quantified using a One Step SYBR PrimeScript RT-PCR Kit II (Takara) and the following dengue group-specific primers: DN-F, 5′-CAATATGCTGAAACGCGAGAGAAA-3′, and DN-R, 5′-CCCCATCTATTCAGAATCCCTGCT-3′ (Phanthanawiboon et al., 2016). The reaction conditions were as follows: 50°C for 30 min, 95°C for 15 min, and then 40 cycles of 95°C for 20 s, 55°C for 30 s, and 72°C for 30 s, followed by a melting curve analysis step. PCR was performed using a LightCycler 96 (Roche). The quantity of vRNA in the initial total RNA was determined by interpolation analysis from a standard curve generated from 10-fold serial dilutions of *in vitro*-transcribed DENV-2 R05-624 RNA prepared using the MEGAscript Kit (Ambion) (Phanthanawiboon et al., 2016).

### 2.9 Protection analysis

Mice were injected intraperitoneally with 100 μg of a purified, functional grade anti-mouse TNF-α antibody (Ab) (XT3.11; Bio X Cell) or 100 μg of an isotype control Ab (MOPC-21; Bio X Cell), on the first day p.i.

### 2.10 Flow cytometry analysis

The small intestine from the duodenum to the small intestine-cecum junction was removed on day 4 p.i. Mesenteric fat was removed, and the intestine was opened longitudinally, washed in PBS to remove fecal matter, and shaken for 20 min at 37°C in HBSS (WAKO) containing 5 mM EDTA. After removing epithelial cells and fat tissue, single cell suspensions were produced by mincing the tissues and incubating them for 1 h at 37°C (with agitation) with 4 mL RPMI1640 (SIGMA) containing 4% bovine serum albumin (BSA) (WAKO), 1 mg/mL collagenase type 2 (Worthington Biochemical Corporation), 1 mg/mL dispase II (WAKO), and 40 μg/mL DNase I (Roche). Digested tissues were filtered through a 70 μm filter and washed with RPMI1640. The resulting cells were pelleted and washed with 20 mL HBSS supplemented with 5 mM EDTA. The cells were resuspended in 5 mL 30% Percoll (GE Healthcare), overlaid onto 4 mL 80% Percoll, and centrifuged at 1,200 × g for 30 min. Isolated lamina propria cells were collected from the Percoll gradient interface and washed with RPMI1640. For FACS analysis, cells were resuspended in FACS buffer (HBSS supplemented with 0.5% BSA), incubated at 4°C for 10 min with TruStain FcX anti-mouse CD16/32 antibody (BioLegend), and then stained at 4°C for 30 min with the following fluorochrome-conjugated antibodies: CD45 (30-F11, BD), CD11b (M1/70, BioLegend), and Ly6G (1A8, BioLegend). Stained cells were analyzed using a FACSLyric cytometer (BD Biosciences), and data were analyzed using FlowJo software (BD Biosciences).

### 2.11 Data analysis

In all the bar graphs and scatter plots, data are expressed as mean ± standard error of the mean (SEM). All data were analyzed using the GraphPad Prism software (GraphPad, San Diego, CA, United States). Statistical analysis was performed using one-way ANOVA, and the significance of differences was assessed using Tukey’s multiple comparison test for multiple comparisons. Comparisons between two groups were performed using two-tailed student’s *t*-test. For the graphs of IL-6 mRNA, MMP-8 mRNA, MMP-3 mRNA, and vRNA, log-transformed data were used for statistical analysis. Survival data were evaluated using the log-rank (Mantel–Cox) test. A *p*-value of <0.05 was considered statistically significant; ^*^p < 0.05, ^**^p < 0.01, ^***^p < 0.001, and ^****^p < 0.0001. Details of the sample sizes are included in the figure legends.

## 3 Results

### 3.1 Adaptation of DENV-1 and DENV-3 in murine organs

We have previously reported that DV3P12/08 clinical isolate causes lethal infection with severe dengue symptoms in IFN-α/β/γR KO mice (Phanthanawiboon et al., 2016; Kurosu et al., 2023b). Some, but not all, DENVs are lethal in IFN-α/β/γR KO mice, some of which show symptoms of severe dengue, such as vascular leakage (Phanthanawiboon et al., 2016). To search for virulent DENV in mice, we examined lethality of several DENV-1s in IFN-α/β/γR KO mice and found that DV1-5 clinical isolate caused acute death (Supplementary Table S1). Besides, IFN-α/β/γR KO mice infected with DV1-5 showed clear gross changes in the liver and intestine, i.e., color fade of the liver and small intestine swelling (data not shown), similar to DV3P12/08-infected IFN-α/β/γR KO mice (Phanthanawiboon et al., 2016; Kurosu et al., 2023b). We then examined if DV1–5 or DV3P12/08 causes lethal infection in IFN-α/βR KO mice singly deficient in the IFN-α/β receptor. However, neither original DV1–5 nor DV3P12/08 caused lethal infection in IFN-α/βR KO mice (data not shown). Therefore, in anticipation of viral adaptation to IFN-α/βR KO mice, we passaged both viruses in IFN-α/βR KO mice, respectively, by targeting specific organs (Figure 1A). The DV1-5P7Sp, passaged seven times in the spleen of IFN-α/βR KO mice, demonstrated lethality (Table 1). In contrast, DV1-5P3Ser, which was passaged in the serum of IFN-α/βR KO mice, showed no lethality. DV3P12/08P4Bm and DV3P12/08P4Liver, passaged four times in the bone marrow and liver of IFN-α/βR KO mice, respectively, demonstrated lethality. DV1-5P7Sp and DV3P12/08 were selected for further analyses. We then challenged these viruses in LysM Cre^+^*Ifnar*^flox/flox^ mice. Intravenous infection with parental DV1–5 or parental DV3P12/08 did not result in mortality in LysM Cre^+^*Ifnar*^flox/flox^ mice (Figures 1B,C). However, intravascular infection with even low amounts of DV1-5P7Sp (1.0 × 10^4^ FFU/mouse) demonstrated 100% lethality in LysM Cre^+^*Ifnar*^flox/flox^ mice (Figure 1D), and that of DV3P12/08P4Bm caused lethal infection (Figure 1E). Intraperitoneal infection of LysM Cre^+^*Ifnar*^flox/flox^ mice with DV1-5P7Sp or DV3P4Bm (3.6 × 10^7^ FFU/mouse) caused lethal infection, while their parental viruses did not (Figures 1D,E).

### 3.2 Growth appearances of LysM Cre^+^*Ifnar*^flox/flox^ mice infected with DV1-5P7Sp or DV3P12/08P4Bm and vascular leakage in the liver and small intestine

**Figure 2:**
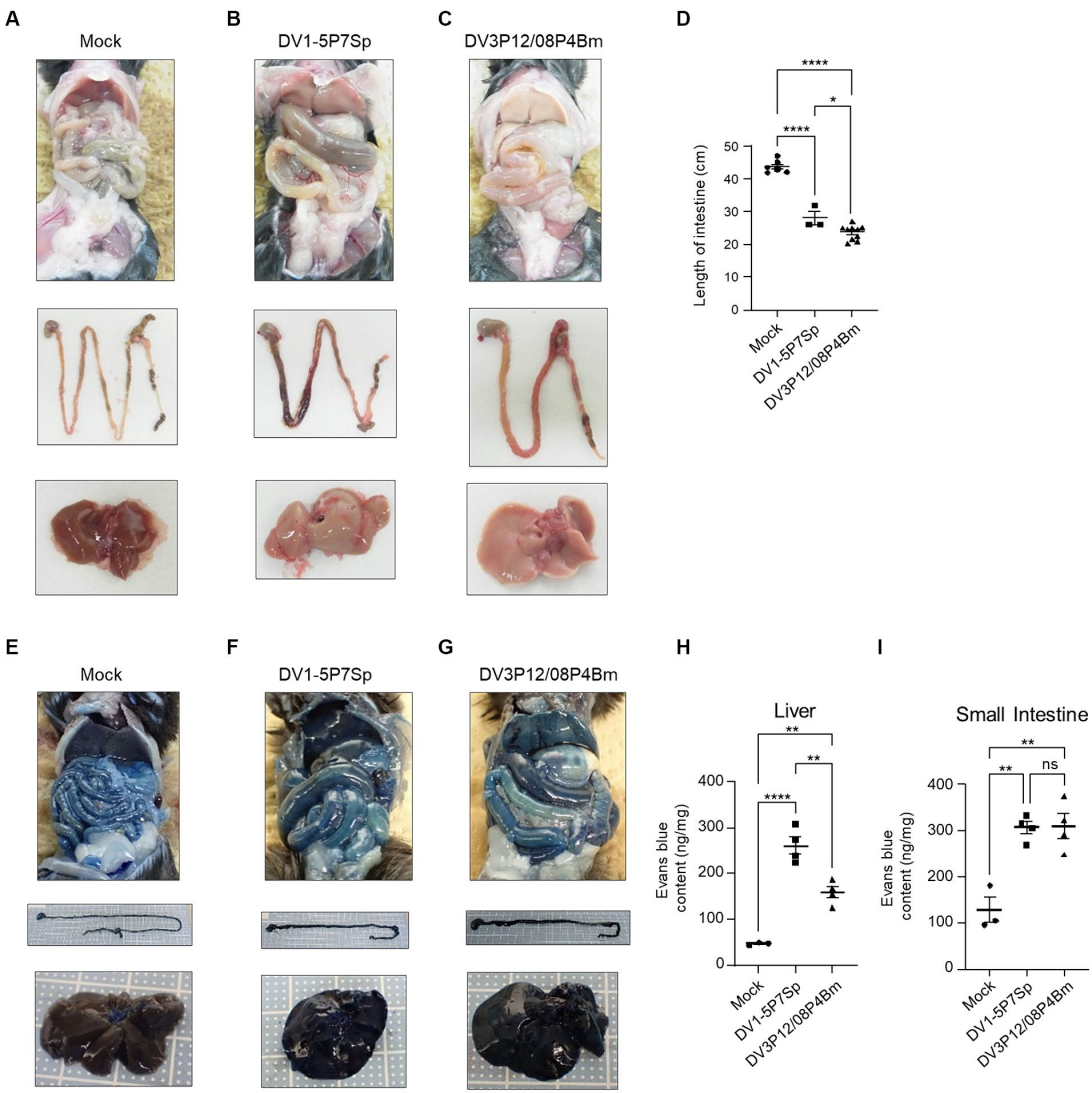
Gross appearance of LysM Cre + Ifnar^flox/flox^ mice. **(A–C)** Ventral (top), intestine (middle), and liver (bottom) gross appearances of LysM Cre + Ifnar^flox/flox^ **(A)** mock-infected, **(B)** DV1-5P7Sp, or **(C)** DV3P12/08P4BM-infected mice. The LysM Cre + Ifnar^flox/flox^ mice (10–12 weeks old) were intravenously infected with 1.0 × 10^5^ focus-forming units (FFU) DV1-5P7Sp or 3.6 × 10^7^ FFU DV3P12/08P4Bm and sacrificed at day 4 post-infection (p.i.). **(D)** The lengths of the intestines from stomach to rectum were measured. **(E–G)** Extravasation of Evans Blue into the liver and small intestines of **(E)** mock-, **(F)** DV1-5P7Sp-, or **(G)** DV3P12/08- infected LysM Cre + Ifnar^flox/flox^ mice. Ventral (top), intestine (middle), and liver (bottom). **(H,I)** Quantification of Evans Blue in the liver **(H)** and small intestine **(I)** of mock-, DV1-5P7Sp-, or DV3P12/08-infected LysM Cre + Ifnar^flox/flox^ mice. Data were analyzed using one-way ANOVA and significance was assessed by Tukey’s multiple comparison test. ^*^p < 0.05, ^**^p < 0.01, ^***^p < 0.001, and ^****^p < 0.0001.

In a previous model of DV3P12/08-infected IFN-α/β/γR KO mice, we observed clear gross changes in the liver and small intestine, i.e., color fade of the liver and swollen of the small intestine (Kurosu et al., 2023b). LysM Cre^+^*Ifnar*^flox/flox^ mice infected with DV1-5P7Sp or DV3P12/08P4Bm showed a faded liver color on day 4 p.i. with faded DV1-5P7Sp or with DV3P12/08P4Bm (Figures 2A–C). The intestines of the mice infected with both viruses individually were swollen (Figures 2B,C) and shortened (Figure 2D). These features suggest possible pathological damage and vascular leakage in these organs. Vascular leakage was evaluated by injecting Evans Blue. Infection with both viruses individually increased vascular leakage in both the liver and small intestine (Figures 2E–I). DV1-5P7Sp appeared to induce more severe vascular leakage in the liver than DV3P12/08P4Bm (Figure 2H).

### 3.3 Histological examination of organs harvested from infected mice

**Figure 3:**
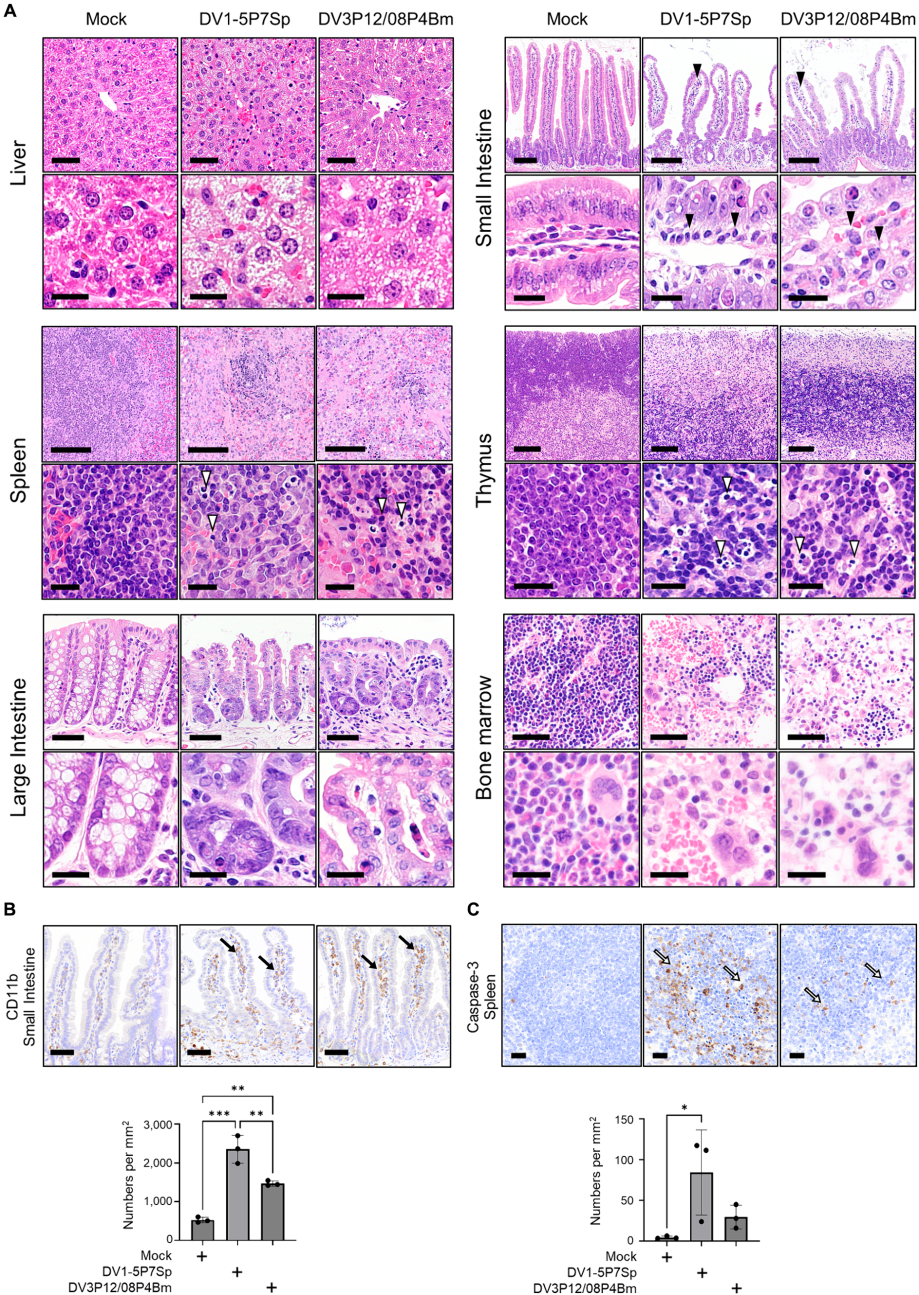
Histopathological examination of tissues of LysM Cre + Ifnar^flox/flox^ mice infected with DV1-5P7Sp or DV3P12/08P4BM. LysM Cre + Ifnar^flox/flox^ mice were intravenously infected with 1.0 × 10^5^ focus-forming units (FFU) DV1-5P7Sp or 3.6 × 10^7^ FFU DV3P12/08P4Bm and sacrificed at day 4 post-infection (p.i.). **(A)** Sections of the liver, small intestine, spleen, thymus, large intestine, and bone marrow were prepared, stained with hematoxylin and eosin, and observed under low (×40) and high (×400) magnification. Bars = 50 μm (upper) and 20 μm (lower) in the liver, 250 μm (upper) and 20 μm (lower) in the small intestine, 100 μm (upper) and 20 μm (lower) in the spleen, 100 μm (upper) and 20 μm (lower) in the thymus, 50 μm (upper) and 20 μm (lower) in the large intestine, and 50 μm (upper) and 20 μm (lower) in the bone marrow. The closed arrowheads indicate infiltrating cells and the open arrowheads indicate apoptotic cells. **(B)** Intestinal sections were subjected to immunostaining with anti-CD11b Ab. The closed arrows indicate infiltrated CD11b-positive cells. Bar = 50 μm. **(C)** Spleen sections were subjected to immunostaining with anti-caspase-3 Ab. The open arrows indicate apoptotic cells. Bar = 20 μm. Cell numbers were analyzed by one-way ANOVA. Significance was assessed by Tukey’s multiple comparison test. ^*^p < 0.05, ^**^p < 0.01, ^***^p < 0.001, and ^****^p < 0.0001. The results are expressed as the mean ± SEM. The experiment was repeated three times with similar results.

Next, we performed histological analysis of H&E-stained sections from the organs of LysM Cre^+^*Ifnar*^flox/flox^ mice. The livers of mice infected with DV1-5P7Sp and DV3P12/08P4Bm showed edematous changes and swollen hepatocytes (Figure 3A). In the small intestines of mice infected with both viruses, edematous changes and infiltration of inflammatory cells were observed. The number of infiltrating CD11b-positive cells was increased by infection (Figure 3B). The spleens of mice infected with both viruses showed apoptotic lymphocytes with a marked decrease in lymphocytes in the white pulp. The increased number of apoptotic cells was examined by caspase-3 staining (Figure 3C). The above observations were quite similar to those in the previous DV3P12/08-infected IFN-α/β/γR KO mouse model (Phanthanawiboon et al., 2016). In the thymus, there were many apoptotic cells, tingible body macrophages, and a massive loss of cortical lymphocytes, suggesting damage to the thymus. The large intestines of mice infected with both viruses showed apoptotic epithelial cells and infiltrated immune cells. The bone marrow showed a drastic reduction in hematopoietic cells, suggesting bone marrow suppression, which has been observed in dengue patients (La Russa and Innis, 1995).

### 3.4 Virus titers in organs

**Figure 4:**
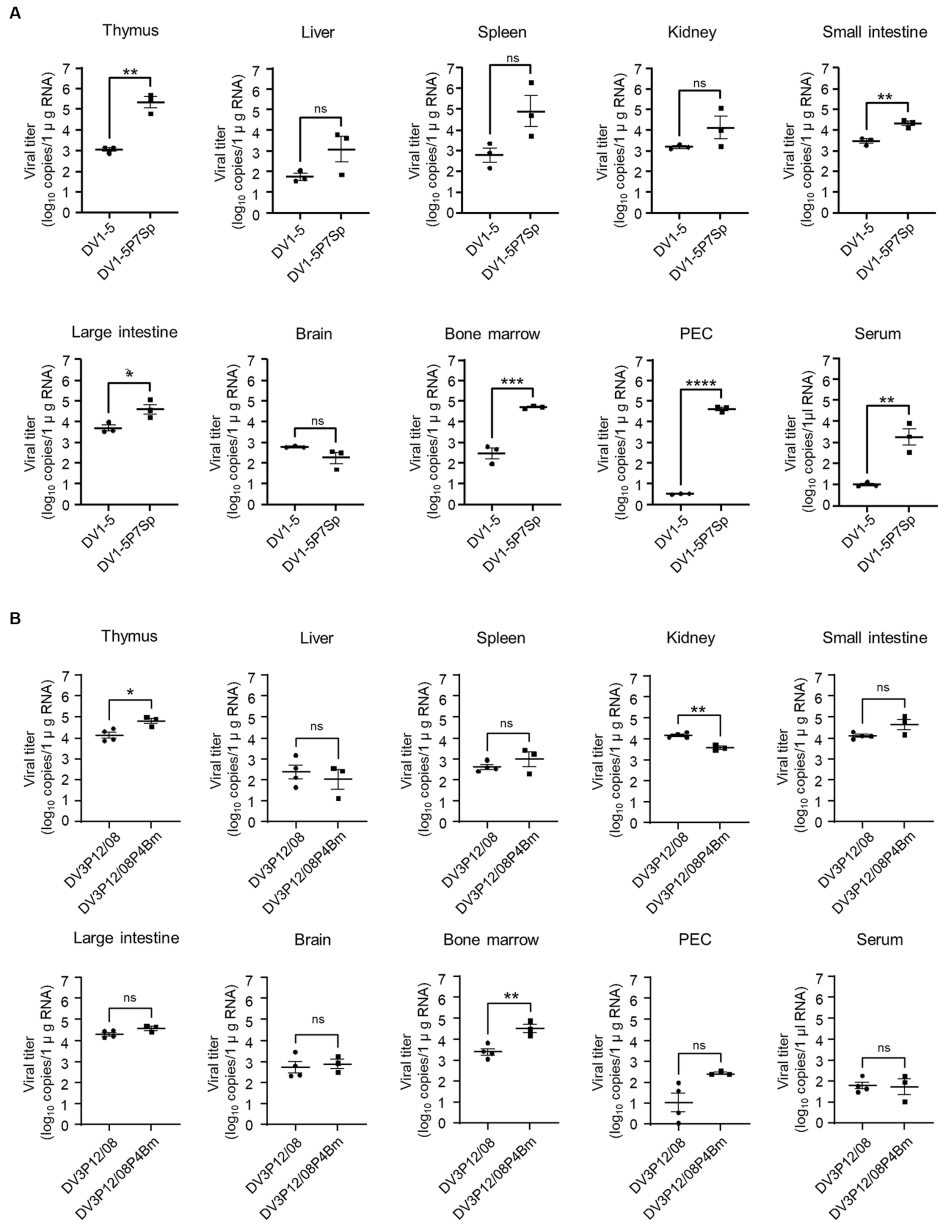
Virus distribution in organs. LysM Cre + Ifnar^flox/flox^ mice were intravenously infected with **(A)** 1.0 × 10^5^ focus-forming units (FFU) DV1-5P7Sp or **(B)** 3.6 × 10^7^ FFU DV3P12/08P4Bm and sacrificed at day 4 (DV1-5P7Sp) or 3 (DV3P12/08P4Bm) post-infection. Viral titers in the small intestine, liver, spleen, thymus, kidney, peritoneal exudate cells (PEC), and bone marrow were measured by qRT-PCR. The results are expressed as the mean ± SEM. Each symbol represents an individual mouse. Data were analyzed by one-way ANOVA, and significance was assessed by Turkey’s multiple comparison test. ^*^p < 0.05, ^**^p < 0.01, ^***^p < 0.001, and ^****^p < 0.0001.

The next question was whether the adapted viruses could improve viral production in mice. Virus titers in each organ of LysM Cre^+^*Ifnar*^flox/flox^ mice infected with adapted viruses were compared with those infected with their parental viruses. DV1-5P7p increased virus production in the thymus (2.9 × 10^5^ vs. 1.2 × 10^3^ FFU/mL), small intestine (2.2 × 10^4^ vs. 3.2 × 10^3^ FFU/mL), large intestine (5.5 × 10^4^ vs. 5.5 × 10^3^ FFU/mL), bone marrow (5.2 × 10^4^ vs. 3.9 × 10^2^ FFU/mL) and PEC (3.5 × 10^3^ vs. 1.0 × 10^1^ FFU/mL) (Figure 4A). DV1-5P7Sp also significantly increased the serum virus titer (4.3 × 10^4^ vs. 3.1 × 10^0^ FFU/mL). Contrarily, DV3P12/08P4Bm showed higher virus production only in the thymus (6.7 × 10^4^ vs. 1.5 × 10^4^ FFU/mL) and bone marrow (3.8 × 10^4^ vs. 3.0 × 10^3^ FFU/mL) (Figure 4B). Both viruses grew well in the thymus and bone marrow, and DV1-5P7Sp appeared to increase viral production more efficiently than DV3P12/08P4Bm.

### 3.5 Replication of the parental DENV and the adapted virus in Vero cells

**Figure 5:**
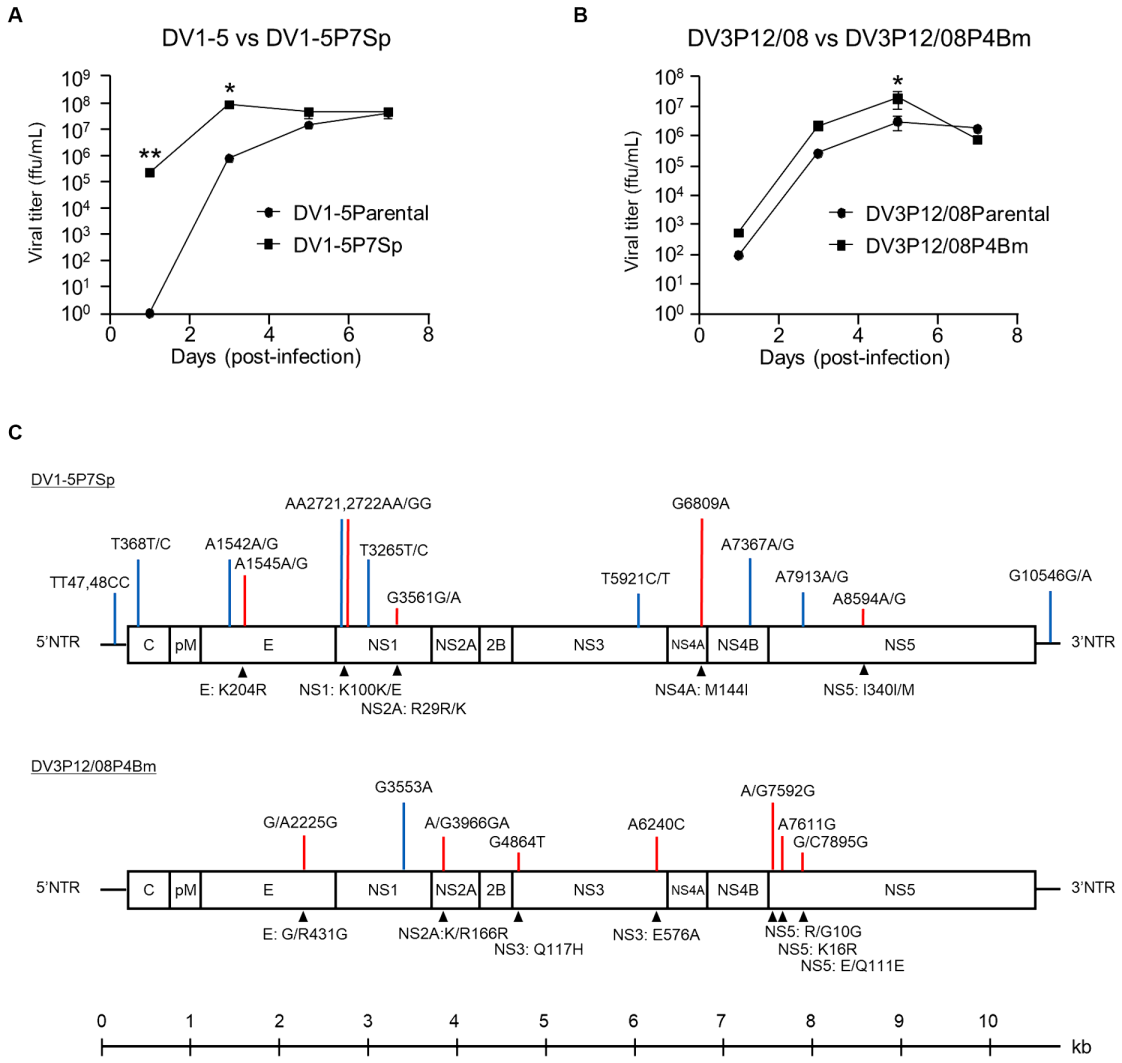
Replication of **(A)** DV1-5, DV1-5P7Sp, **(B)** DV3P12/08, and DV3P12/08P4Bm in Vero cells. Vero cells were infected with DV1-5, DV1-5P7Sp, DV3P12/08, and DV3P12/08P4Bm multiplicity-of-infection (MOI) at 0.01. Culture media were collected at indicated time points post-infection. Viral titers were determined by focus-forming assays and expressed as the logarithm of focus-forming units (FFU) per milliliter. Results are expressed as mean ± SD of triplicate experiments. Viral titers of each day were analyzed using the unpaired *t*-test after log transformation. ^*^p < 0.1 and ^**^p < 0.01. **(C)** Diagram of the DV1-5P7Sp and DV3P12/08P4Bm genomes. DV1-5P7Sp has fourteen substitutions compared with the parental DV1-5 strain shown in the upper part of the genome diagram. P12/08P4Bm has eight substitutions. Blue bars, synonymous substitutions; red bars, nonsynonymus substitutions. Lower highlights (triangles) show the amino acid substitutions. Position numbers are relative to those in the reference DV1-5 or DV3P12/08 strain. The numbers indicate the amino acid position in each viral protein. Single-nucleotide polymorphisms are present at 368, 1,542, 1,545, 2,721, 2,722, 3,265, 3,561, 5,921, 7,367, 7,912, and 8,594 in DV1-5P7Sp. NTR, non-coding region; C, capsid; pr, premembrane; M, membrane; E, envelope; NS, non-structural protein.

The replication of DV1-5P7Sp and DV3P12/08P4Bm was compared to that of their parental viruses in Vero (African green monkey kidney) cells. DV1-5P7Sp showed higher virus production (2.1 × 10^5^ FFU/mL) at day 1 p.i. and reached maximum virus production (8.3 × 10^7^ FFU/mL) at day 3 p.i. (Figure 5A), whereas DV1–5 showed no detectable virus production at day 1 p.i. and lower production at day 3 p.i. (7.3 × 10^5^ FFU/mL). At 5 and 7 days p.i., the virus titers of both viruses were similar (1.4 × 10^7^ and 4.3 × 10^7^ FFU/mL at day 5 p.i., 4.0 × 10^7^ and 4.5 × 10^7^ FFU/mL at day 7 p.i.). On the other hand, DV3P12/08P4Bm and DV3P12/08 started increasing from day 1 p.i. (5.3 × 10^2^ and 9.2 × 10^1^ FFU/mL, respectively) and reached peak titers at day 5 p.i. (1.9 × 10^7^ and 3.0 × 10^6^ FFU/mL, respectively) (Figure 5B). DV3P12/08P4Bm increased in a manner similar to that of DV3P12/08, although the DV3P12/08P4Bm titer was slightly higher than that of DV3P12/08 at day 5 p.i. (2.1 × 10^6^ vs. 2.6 × 10^5^ FFU/mL, respectively). Collectively, DV1-5P7Sp showed faster replication ability, even in Vero cells.

### 3.6 Sequence analysis of adapted viruses and their original strains

Sequence analysis was performed to determine substitutions in the adapted viruses. DV1-5P7Sp had 14 nucleotide substitutions in the 5′NTR, envelope, NS1, NS3, NS4A, NS4B, NS5, and 3′NTR genes, and 5 amino acid substitutions in E, NS1, NS4A, and NS5 (Figure 5C). Some substitutions included two types of nucleotides, indicating quasispecies. In contrast, DV3P12/08P4Bm had eight nucleotide substitutions in the E, NS1, NS2A, NS3, and NS5 genes and 7 amino acid substitutions in E, NS2A, NS3 and NS5 (Figure 5C).

### 3.7 Protection of mice from lethal infection by blockade of TNF-α signaling

**Figure 6:**
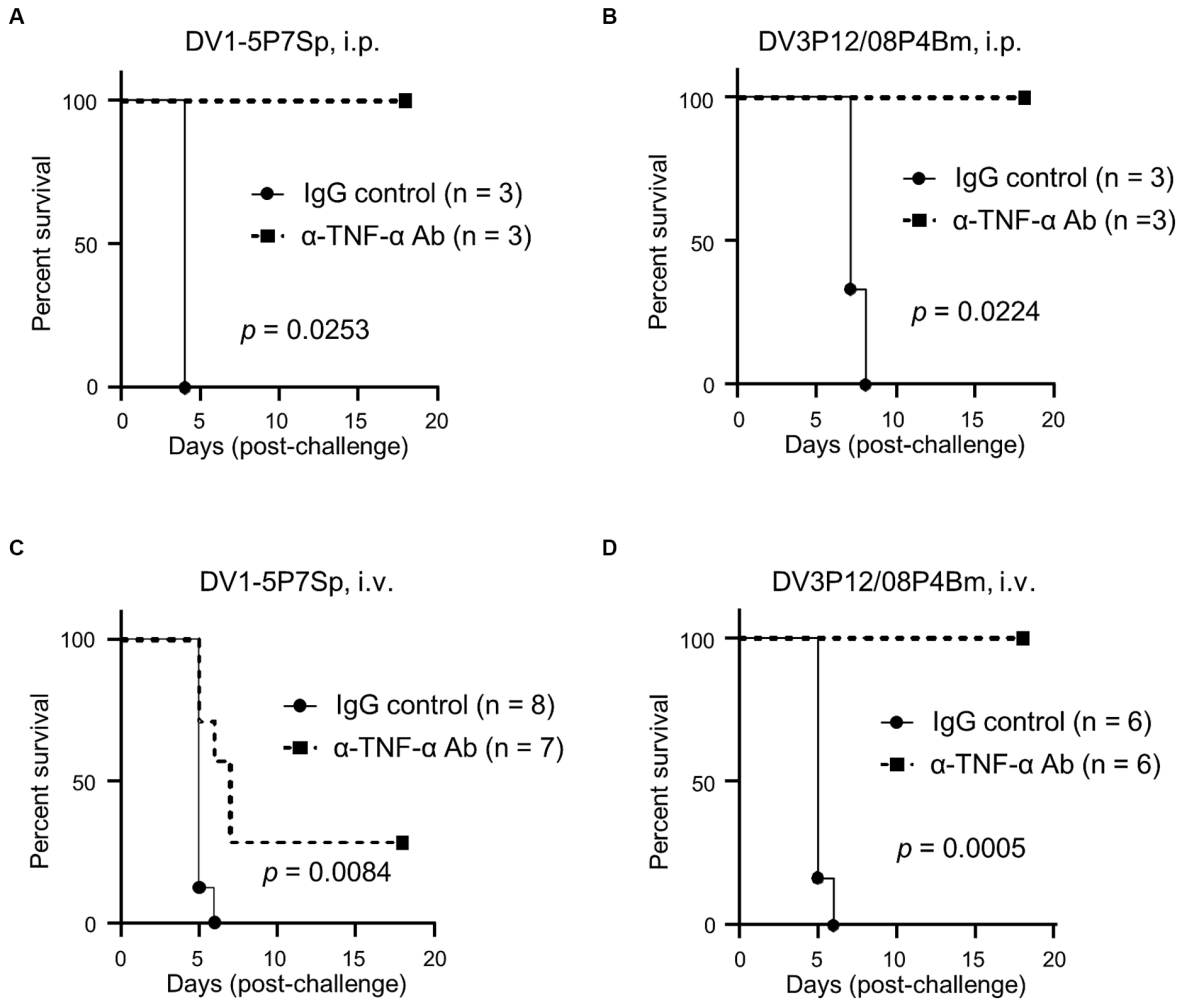
Anti-TNF-α Ab treatment. **(A,B)** LysM Cre + Ifnar^flox/flox^ mice were intravenously infected with 1.0 × 10^5^ focus-forming units (FFU) DV1-5P7Sp or 1.8 × 10^7^ FFU DV3P12/08P4Bm and intraperitoneally injected with 0.5 mg α-TNF Ab or control normal IgG at day 1 p.i., and survival was monitored. Kaplan–Meier survival curves show the percentage of mice surviving at the specified days post-infection. **(C,D)** LysM Cre + Ifnar^flox/flox^ mice were intraperitoneally infected with 6.0 × 10^5^ FFU DV1-5P7Sp and intraperitoneally injected with 0.5 mg α-TNF Ab or control normal IgG at day 1 p.i., and survival was monitored. Statistical differences were evaluated by the Log-rank (Mantel–Cox) test. Percent body weight change of infected mice from the anti-TNF-α Ab-injected (solid circles) versus IgG-injected (solid squares) groups are presented.

In previous mouse models for severe dengue, blockade of TNF-α protected mice from lethal infection (Atrasheuskaya et al., 2003; Shresta et al., 2006; Phanthanawiboon et al., 2016; Kurosu et al., 2023b), suggesting a central role in lethality. When LysM Cre^+^*Ifnar*^flox/flox^ mice were intraperitoneally infected, anti-TNF-α Ab treatment protected all mice from lethal infection with DV1-5P7Sp (Figure 6A). On the other hand, anti-TNF-α Ab treatment completely protected LysM Cre^+^*Ifnar*^flox/flox^ mice from intraperitoneal lethal infection with DV3P12/08P4Bm (Figure 6B). However, anti-TNF-α Ab only partially protected LysM Cre^+^*Ifnar*^flox/flox^ mice from DV1–5 upon its intravenous injection (Figure 6C). Contrarily, anti-TNF-α Ab completely protected LysM Cre^+^*Ifnar*^flox/flox^ mice even when DV3P12/08P4Bm intravenously injected (Figure 6D). Although the routes of infection affected protective efficacy of anti-TNF-α Ab treatment, these results suggest that the TNF-α signaling pathway is strongly implicated in the pathogenesis of these models.

### 3.8 Expressions of IL-6, MMP-8, and MMP-3

**Figure 7:**
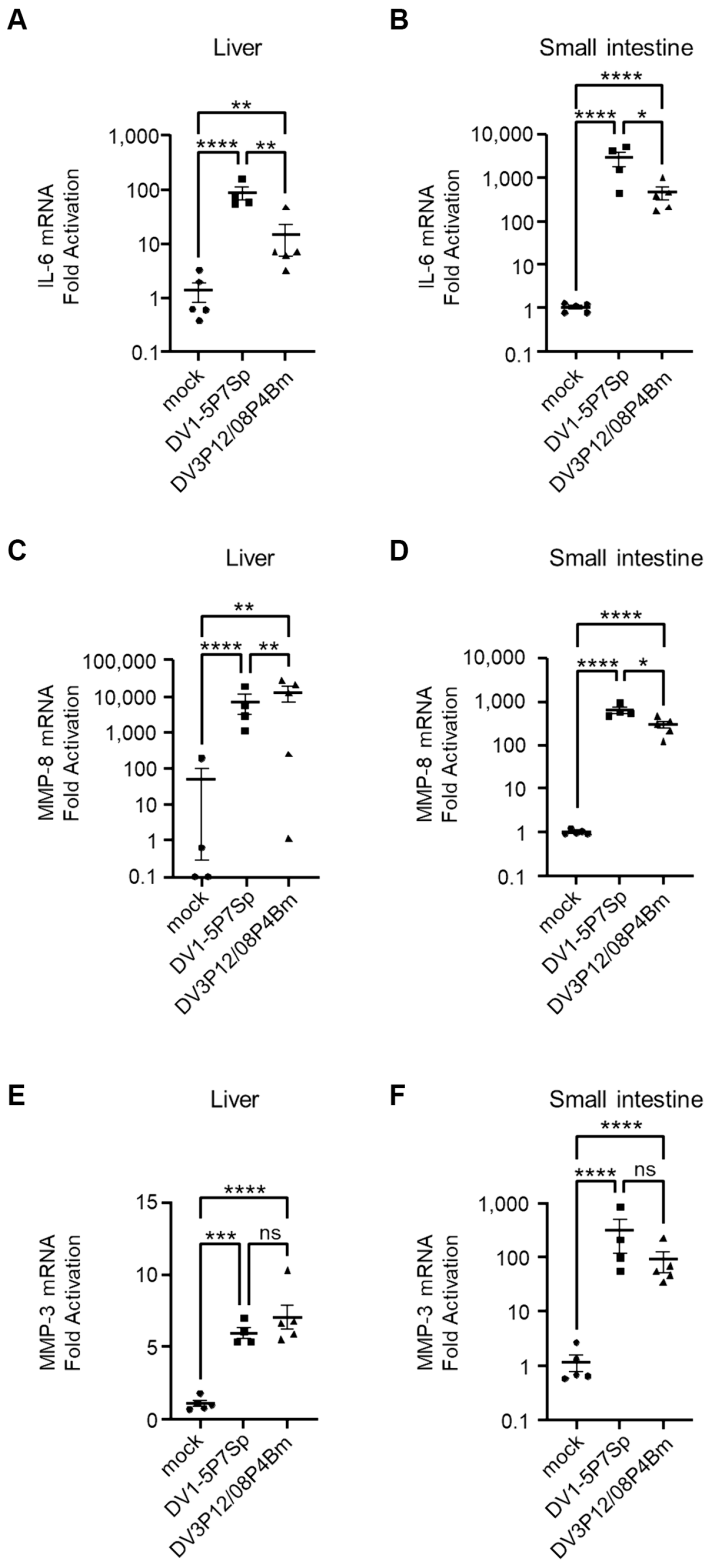
Levels of IL-6, MMP-8, and MMP-3 in the liver and small intestine. LysM Cre + Ifnar^flox/flox^ mice (8–10 weeks old) were intravenously infected with 1.0 × 10^5^ focus-forming units (FFU) DV1-5P7Sp or 1.8 × 10^7^ FFU DV3P12/08P4Bm. Total RNA was extracted from the liver (*n* = 4–5) and small intestine (*n* = 4–5) at day 4 post-infection (p.i.) and subjected to quantitative RT-PCR. The expression of each mRNA was calculated relative to that in mock-infected mice. Levels of mRNA expression of IL-6 **(A,B)**, MMP-8 **(C,D)**, and MMP-3 **(E,F)** in the liver **(A,C,E)** and small intestine **(B,D,F)** are presented. Data were analyzed using one-way ANOVA, and the significance of differences was assessed by Tukey’s multiple comparison test. ^*^p < 0.05, ^**^p < 0.01, ^***^p < 0.001, and ^****^p < 0.0001.

Vascular leakage, driven by a cytokine storm, occurs in two steps: a cytokine event and an effector event. TNF-α is an upstream regulator of many cytokines and is a key regulator in this model (Figures 6A–D). We have previously reported very high levels of IL-6 induction, which is considered a key cytokine linking both cytokine and effector events, leading to vascular leakage (Kurosu et al., 2023b). Therefore, we measured IL-6 mRNA expression in the liver and small intestine using qRT-PCR. Both DV1-5P7Sp and DV3P12/08P4Bm infections increased IL-6 mRNA in the liver (8.9 × 10 or 1.4-fold) and small intestine (2.8 × 10^2^ or 4.5× 10-fold) (Figures 7A,B). The increase of IL-6 mRNA in the small intestine of LysM Cre^+^*Ifnar*^flox/flox^ mice infected with DV1-5P7Sp or DV3P12/08P4Bm was much higher than that in the liver. Compared to the increase in IL-6 mRNA by DV3P12/08P4Bm, that by DV1-5P7Sp was higher in both the liver and small intestine. We also observed higher induction of matrix metalloprotease-8 and -3, which are thought to be important effectors responsible for vascular leakage (Kurosu et al., 2023b). Infection with DV1-5P7Sp or DV3P4Bm increased MMP-8 mRNA (7.2 × 10^3^ or 1.3 × 10^4^-fold) in the liver (6.5 × 10^2^ or 3.0 × 10^2^-fold) and small intestine (Figures 7C,D). Furthermore, these infections increased MMP-3 mRNA levels in the liver (6.0 or 7.1-fold) and small intestine (3.1 × 10^2^ or 9.1× 10-fold), although only subtle increases were observed in the liver (Figures 7E,F). These results suggest that the current model is similar to the previous model in terms of downstream events following a cytokine storm.

### 3.9 Neutrophil invasion into the small intestine of LysM Cre^+^*Ifnar*^flox/flox^ mice following infection with DV1-5P7Sp and DV3P12/08P4Bm

**Figure 8:**
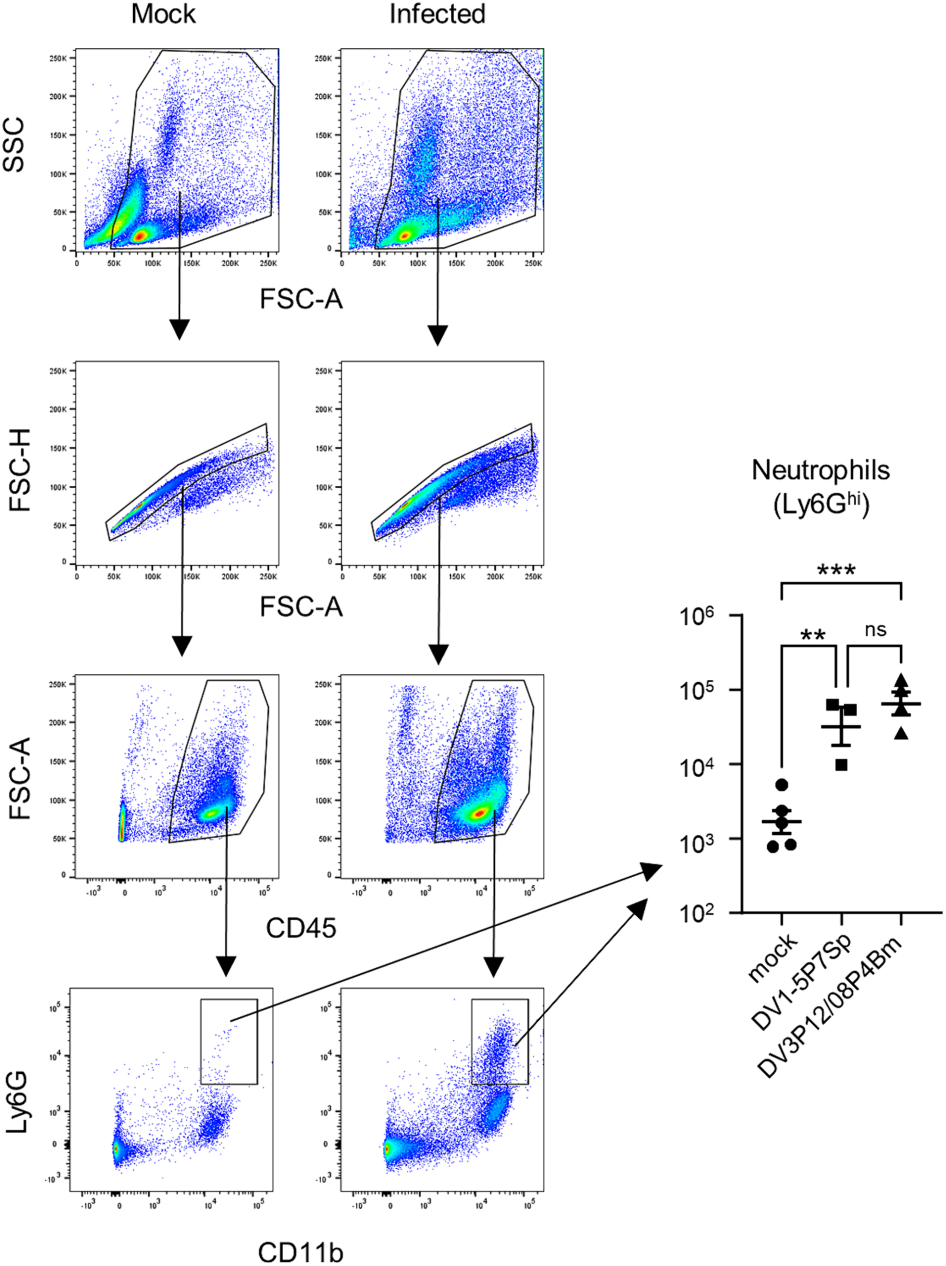
Analysis of infiltrated neutrophils and monocytes-macrophages in the small intestine of infected mice. Representative flow cytometry plots of gated intestinal cells. LysM Cre + Ifnar^flox/flox^ mice (8–10 weeks old) were intravenously infected with 1.0 × 10^5^ focus-forming units (FFU) DV1-5P7Sp or 1.8 × 10^7^ FFU DV3P12/08P4Bm and sacrificed under anesthesia at day 4 post-infection (p.i.), and the small intestine was collected. The SSC-FSC-A profile was used to distinguish leukocyte populations from other cell populations. Duplets were removed by singlet gating, and debris was removed from the analysis. Leukocyte populations were further gated by FSC-A-CD45 analysis. CD45^+^ leukocyte populations were gated by Ly6G-CD11b analysis, and neutrophils were classified as CD11b^+^Ly6G^hi^. The total numbers of CD11b^+^Ly6G^hi^ neutrophils were quantified. Each symbol represents an individual mouse. Cell numbers were analyzed by one-way ANOVA. Significance was assessed by Tukey’s multiple comparison test. ^**^p < 0.01, ^***^p < 0.001, and ^****^p < 0.0001. The results are expressed as the mean ± SEM. The experiment was repeated two times with similar results.

MMP-8 was suspected to be the most important effector that leads to vascular leakage because it was found to be increased commonly in the liver and small intestine of DV3P12/08-infected IFN-α/β/γR KO mice (Kurosu et al., 2023a). Neutrophils are considered a major source of MMP-8. We also previously reported the high level of neutrophil infiltration in the small intestine of DV3P12/08-infected IFN-α/β/γR KO mice and the corresponding value for pathogenesis (Kurosu et al., 2023b). When we examined the infiltrating cells in the small intestine of LysM Cre^+^*Ifnar*^flox/flox^ mice infected with DV1-5P7Sp or DV3P12/08, we observed significantly increased neutrophil numbers in the small intestine (Figure 8). We confirmed the involvement of neutrophils in the pathogenesis of this disease.

## 4 Discussion

Several groups, including ours, have analyzed the pathogenesis of severe dengue using AG129 or IFN-α/β/γR KO mice (Shresta et al., 2006; Watanabe et al., 2015; Kurosu et al., 2023b). In these models, a lethal infection with vascular leakage, which is the physiological basis of severe dengue, was observed. However, as all cells of IFN-α/β/γR KO mice lack both type I and type II IFN-R genes, these models have been criticized as not reflecting actual human pathogenesis. Therefore, we aimed to create a mouse model that was as close as possible to immunocompetent mice. The LysM Cre^+^*Ifnar*^flox/flox^ mice used in this study lacked type I IFN-R in the myeloid lineage cells (Clausen et al., 1999; Pinto et al., 2015). Macrophages, monocytes, and dendritic cells, which are myeloid cells, are considered the targets of DENV in humans (Rothman, 2011). The DENV clinical isolates we examined were not lethal to LysM Cre^+^*Ifnar*^flox/flox^ mice, even under ADE conditions in the current study (Figure 1; Supplementary Figure S1). This is probably because the original clinical isolates were less virulent than DENV-2 D2S20 (Pinto et al., 2015). DENV-2 D2S20 was produced by passage in mice several times (Makhluf et al., 2013). On the other hand, we successfully obtained virulent DV1-5P7Sp and DV3P12/08P4Bm, which are highly lethal and cause vascular leakage, via organ-by-organ passaging in the spleen and bone marrow, respectively. The reason for the higher virulence of these viruses is unclear; however, one possible reason is their higher replicative ability. Notably, both viruses showed higher proliferative capacity in the thymus and bone marrow (Figures 5A,B), although DV1-5P7Sp grew relatively efficiently in most organs. In LysM Cre^+^*Ifnar*^flox/flox^ mice, myeloid cells are the target of infection with DV1-5P7Sp and DV3P12/08P4Bm, suggesting that the infection of myeloid cells in the thymus and bone marrow is important or necessary for severe conditions with vascular leakage.

Importantly, blockade of TNF-α signaling protected LysM Cre^+^*Ifnar*^flox/flox^ mice (Figures 6A–D), albeit only partially when mice were intravenously infected with DV1-5P7Sp (Figure 6C). There are two possible reasons for this partial protection. First, DV1-5P7Sp is highly adapted and has a faster propagation ability than DV3P12/08P4Bm (Figures 5A,B). Second, the virus spreads faster to organs when it is administered intravenously than when it is administered intraperitoneally. In the current model, the mice died approximately 5 days p.i. This is the period during which the host immunity begins to function effectively. We previously observed that neutralizing antibodies exist by day 6 p.i. using a similar mouse model with IFN-α/β/γR KO mice (Ramadhany et al., 2015). We established that LysM Cre^+^*Ifnar*^flox/flox^ mice died by cytokine storm induced by DV1-5P7Sp infection or DV3P12/08P4Bm; however, actual protection by anti-TNF-α Ab seemed to be due to prolonging the survival period until mice produced enough levels of neutralizing antibodies. This is possibly why anti-TNF-α treatment only partially protected LysM Cre^+^*Ifnar*^flox/flox^ mice intravenously infected with DV1-5P7Sp.

Myeloid cells include granulocytes, monocytes, macrophages, and dendritic cells (Manz and Boettcher, 2014). Except for granulocytes, these cells are thought to be targets of DENV infection in humans (Guzman et al., 2016). Generally, these cells are thought to play a critical role in the cytokine storm syndrome (CSS) (Liu and Zhao, 2018). Myeloid cells have diverse functions that influence all phases of the cytokine storm physiology, from initiation to resolution (cytokine storm syndrome), and function in both directions by enhancing or suppressing cytokine production. The current model suggests that myeloid cell infection may be sufficient to trigger CSS-induced vascular leakage. This suggests that this model is suitable for studying the mechanisms underlying CSS initiated by myeloid cell infection. It was also reconfirmed that IFN-γR was not involved in vascular leakage. The present model will allow for a more focused exploration of pathogenic mechanisms.

Histopathological analysis showed severe bone marrow suppression and a decrease in hematopoietic cells due to infection (Figure 3). In addition to vascular leakage, bone marrow suppression is a characteristic phenomenon in patients infected with the dengue virus (Bierman and Nelson, 1965; Na-Nakorn et al., 1966; La Russa and Innis, 1995). There are also several theories on the potential cause of bone marrow suppression (Srichaikul and Nimmannitya, 2000); however, the exact mechanism remains unclear. The present model may be useful to elucidate not only the mechanism of vascular leakage, but also that of bone marrow suppression.

Several substitutions were detected in the adapted viruses (Figure 4C). At the amino acid level, the substitution sites in the adapted virus were found in the E, NS1, NS4A, and NS5 genes in DV1-5P7Sp, whereas in DV3P12/08P4Bm, they were found in the E, NS2A, NS3 and NS5 genes. These substitutions were suspected to be responsible for the observed increase in viral replication. In particular, DV1-5P7Sp showed increased proliferative potential not only in mice (Figure 4A), but also in Vero cells (Figure 5A). Furthermore, DV1-5P7Sp was 100% lethal, even at much lower infectious virus doses (Figures 1B,C), suggesting that these five substitutions confer strong virulence, which is probably an ability to replicate efficiently. However, no common substitutions were detected between DV1-5P7Sp and DV3P12/08P4Bm. The causes of the increased viral proliferative abilities of DV1-5P7Sp and DV3P12/08P4Bm may not be the same.

In this model, similar to previous models (Kurosu et al., 2023b), we observed severe vascular leakage in the liver and small intestine (Figures 2E–I), neutrophil infiltration in the small intestine (Figure 8), and increased expression of MMP-3 and MMP-8 (Figures 7C–F). In addition, as in the previous model, IL-6 mRNA expression was induced at abnormally high levels in the intestinal tract (Figures 7A,B), suggesting that IL-6 is a key factor linking phenomena at the cytokine and effector levels. In a DV3P12/08-infected IFN-α/β/γR KO mice model, we reported that neutrophils are potential effectors leading to vascular leakage. Although few studies have focused on neutrophils in dengue virus infection, neutrophilia has been reported in patients with severe dengue (Her et al., 2017). The observation of neutrophil infiltration inducing plasma leakage, even though this mouse model was slightly different from the previous model, suggests that neutrophil infiltration is an important factor in CSS-induced vascular leakage in both models.

Secondary infections and severe diseases are also believed to be associated. Mouse ADE models, including ours, are passively immunized with antibodies against DENV E or prM (Zellweger et al., 2010; Watanabe et al., 2015; Kurosu et al., 2020). Currently, no models can reproduce human-like secondary infections. Although secondary infections are the focus of attention in dengue fever, temporary infections can also cause dengue fever and other severe diseases (Khurram et al., 2014). In particular, DENV-1 and DENV-3, unlike DENV-2 and DENV-4, cause disease through primary infection (Nisalak et al., 2003; Anantapreecha et al., 2005). Therefore, studies using transient infection models may be useful for understanding the mechanisms of severe diseases. It is necessary to develop a mouse model for secondary infection; however, it is also crucial to consider an approach to elucidate the pathogenic mechanisms of severe disease from the perspective of primary infection.

In conclusion, a new model using LysM Cre^+^*Ifnar*^flox/flox^ mice and adapted viruses showed a very similar pathology to previous model using IFN-α/β/γR KO mice in primary infection, which imitate human diseases. This model will allow for more cellular and host/viral factor-specific studies to elucidate detailed pathogenic mechanisms.

## Data availability statement

The datasets presented in this study can be found in online repositories. The names of the repository/repositories and accession number(s) can be found in the article/Supplementary material.

## Ethics statement

The animal study was approved by the Institutional Animal Care and Use Committee of the NIID. The study was conducted in accordance with the local legislation and institutional requirements.

## Author contributions

TK: Conceptualization, Data curation, Formal analysis, Funding acquisition, Investigation, Methodology, Project administration, Resources, Software, Supervision, Validation, Visualization, Writing – original draft, Writing – review & editing. YS: Conceptualization, Data curation, Formal analysis, Investigation, Methodology, Validation, Visualization, Writing – review & editing, Writing – original draft. YA: Investigation, Methodology, Writing – review & editing. MSh: Funding acquisition, Methodology, Writing – review & editing. TY: Methodology, Writing – review & editing. SF: Methodology, Writing – review & editing. NN: Data curation, Funding acquisition, Writing – review & editing, Resources. TS: Funding acquisition, Resources, Writing – review & editing. HE: Conceptualization, Writing – review & editing. MSa: Funding acquisition, Resources, Writing – review & editing.

## Footnotes

^1^ https://www.cdc.gov/dengue/healthcare-providers/clinical-presentation.html
