# Supplementary material for: Mice, myeloid cells, and dengue: a new model for unraveling vascular leakage mysteries
Source: Front Microbiol. 2024 Mar 14;15:1367672. doi: 10.3389/fmicb.2024.1367672 (PMC10972876; doi:10.3389/fmicb.2024.1367672)
Supplement: Supplementary file 1 [file Table_1.DOCX]

SUPPLEMENTARY TABLE 1 Challenge of DENV clinical isolates in IFN-α/β/γR KO mice.

| Virus | Titer (FFU) | Number ^a^ | % Survival | Period ^b^ |
| --- | --- | --- | --- | --- |
| DV1-1 | 6.0×10^6^ | 3 | 100 | 13-15 |
| DV1-2 | 1.3×10^6^ | 3 | 33 | 16-21 |
| DV1-3 | 3.6×10^6^ | 3 | 100 | 11-15 |
| DV1-5 | 5.7×10^6^ | 4 | 100 | 5-6 |

^a^ Numbers of mice used for challenge.

^b^ Period indicates the days when mice condition reached the end point.
