# Supplementary material for: Mice, myeloid cells, and dengue: a new model for unraveling vascular leakage mysteries
Source: Front Microbiol. 2024 Mar 14;15:1367672. doi: 10.3389/fmicb.2024.1367672 (PMC10972876; doi:10.3389/fmicb.2024.1367672)
Supplement: Supplementary file 2 [file Table_2.DOCX]

SUPPLEMENTARY TABLE 2 Primers used for full-length DV1-5, DV1-5P7Sp, DV3P12/08, and DV3P12/08P4Bm.

| Name of primer | Sequence |
| --- | --- |
| DV1_fragment I amplification |  |
| P12/08Fw1-24 | AGTTGTTAGTCTACGTGGACCGAC |
| DV1-5_Seq27Rv | ATCATAAACCACAGGGTCCAAATC |
|  |  |
| DV1_fragment II amplification |  |
| DV1-5Fw_6494 | GAGCCTATAGACATGCAATGGAAGAACTGCC |
| DV1-5_ Rv_ver.6 | AGAACCTGTTGATTCAACAGCACCTTCCATTTTCTG |
|  |  |
| DV3_fragment I amplification |  |
| P12/08Fw1-24 | AGTTGTTAGTCTACGTGGACCGAC |
| P12/08_SeqRv5 | ATGACTCTTCCTTCGGGAATGACC |
|  |  |
| DV3_fragment II amplification |  |
| P12/08_SeqFw18 | CTAAAGCCACTCGTGAAGC |
| P12/08 Rv.ver.6 | AGAACCTGTTGATTCAACAGCACCATTCC |
|  |  |
| DV1 sequencing |  |
| DV1-5_Seq 1 (445-467) | TACACGAGGGGGAGAGCCGC |
| DV1-5_ Seq2 (1050-1072) | CAACATTGGACATTGAACTCTTG |
| DV1-5_Seq3 (1624-1645) | TTGGAACAGACAAGATTTGCTG |
| DV1-5_Seq4 (2109-2131) | CAAGAAAGGAAGCAGCATAGGG |
| DV1-5_Seq5 (2665-2687) | TGACATGAAATTCACAGTGGTTG |
| DV1-5_Seq6 (3206-3227) | CAAACGGCAGGGCCGTGGCACC |
| DV1-5_Seq7 (3749-3768) | CTATTCCGCAGACTAACATCCAG |
| DV1-5_Seq8 (4316-4335) | GAAGCAGAACACTCTGGTGC |
| DV1-5_Seq9 (4898-4917) | GCCCTAGATTTTAAACCCGGCAC |
| DV1-5_Seq10 (5466-5484) | GCCACTCCCCCAGGATCAGTG |
| DV1-5_Seq11 (6053-6072) | CCGGAGAGAGAAAAGAGTGCAGC |
| DV1-5_Seq12 (6662-6680) | CTGCTATGGATGGCCAGTGTAGAGC |
| DV1-5_Seq13 (7237-7255) | AAGGACAGCGGCCGGAATAATG |
| DV1-5_Seq14 (7817-7836) | GGTTGTGGAAGAGGTGGCTGGTC |
| DV1-5_Seq15 (8361-8380) | ACATGTGGCAGTGGAACCAGAGG |
| DV1-5_Seq16 (8969-8988) | GGAAAGGCAAAAGGAAGTCGTGC |
| DV1-5_Seq17 (9545-9564) | TTCACTCAGCGGAGTGGAAGGAG |
| DV1-5_Seq18 (10121-10140) | ATAGGCTTAACAGCAAGGGCCAC |
| DV1-5_Seq19 Rv (392-415) | GTGACTTTCCTCTTTCCTGCTTGC |
| DV1-5_Seq20 Rv (1700-1723) | TCAGTTTGTCCATCTTTAGTCTAC |
| DV1-5_Seq21 Rv (2799-2822) | GTGAAAATTCCAAATCCATAATCC |
| DV1-5_Seq22 Rv (3249-3272) | CTGCAACACCATTCATGAATTATC |
| DV1-5_Seq23 Rv (3701-3724) | ATGCCACTAGACTCAATCCAATTG |
| DV1-5_Seq24 Rv (1562-1590) | CTGCTTCTTTGCATGAGCTGTCTTAAATG |
| DV1-5_Seq25 Rv (1746-1769) | AGCTTGAATGAGCCTGTGCACATC |
| DV1-5_Seq26 Rv (5941-5964) | CTCGAAGAGGGCTGGGATAATCCC |
| DV1-5_Seq27 Rv (7201-7224) | ATCATAAACCACAGGGTCCAAATC |
| DV1-5_Seq28 Rv (8010-8035) | CCAATTTTGCATAAAAATTCTGAATC |
| DV1-5_Rv_end1 | AGAACCTGTTGATTCAACAGCACCA |
|  |  |
| DV3 sequencing |  |
| P12/08_SeqFw1 | GATGTTACCAGCAACACTTGC |
| P12/08_SeqFw2 | TAGAGACATGGGCTCTTAGG |
| P12/08_SeqFw3 | ACGTATGTAAGCACACATACG |
| P12/08_SeqFw4 | GGAGCTACAACAGAGACACC |
| P12/08_SeqFw5 | CCCAGTGGTGACCAAGAAGG |
| P12/08_SeqFw6 | CTGGGAGCCGTGGTACAAGC |
| P12/08_SeqFw7 | CATAATAGATGGGCCAAAC |
| P12/08_SeqFw8 | CCAAACGGCAGGACCTTGG |
| P12/08_SeqFw9 | TGCTCCTCCTCTCAGGGC |
| P12/08_SeqFw10 | GCTTTTGCCACTGTGCCAG |
| P12/08_SeqFw11 | GAACATCCTAACAGTGCTC |
| P12/08_SeqFw12 | CGTAGAGCCTGGGAAGAACC |
| P12/08_SeqFw13 | CAGCTGAGATGGAAGAAGC |
| P12/08_SeqFw14 | TGTCCCTAGCATCAAAGCC |
| P12/08_SeqFw15 | CATTAACACACCAGAAGG |
| P12/08_SeqFw16 | CAGAAATAGGAAGAGTGCC |
| P12/08_SeqFw17 | CACTGGCTGCGATAGTAGC |
| P12/08_SeqFw18 | CTAAAGCCACTCGTGAAGC |
| P12/08_SeqFw19 | TCAGTTATCCCGGAAAGAG |
| P12/08_SeqFw20 | GGAGAATCTTCACCAAGC |
| P12/08_SeqFw21 | AGGATCAAGGAGGAGCATAG |
| P12/08_SeqFw22 | AGCTGTTTTCACAGAGGAG |
| P12/08_SeqFw23 | ATGACCTGCACAATGAGG |
| P12/08_SeqFw24 | GACATGGGAAAGGTTAGG |
| P12/08_SeqFw25 | GTCTGGAACAGGGTGTGG |
| P12/08_SeqFw26 | GAGCAAACCGTGCTGCCTG |
| P12/08_SeqRv1 | GGCCGTTCAGCAATCCTC |
