# Supplementary figures and images for: Mice, myeloid cells, and dengue: a new model for unraveling vascular leakage mysteries
Source: Front Microbiol. 2024 Mar 14;15:1367672. doi: 10.3389/fmicb.2024.1367672 (PMC10972876; doi:10.3389/fmicb.2024.1367672)

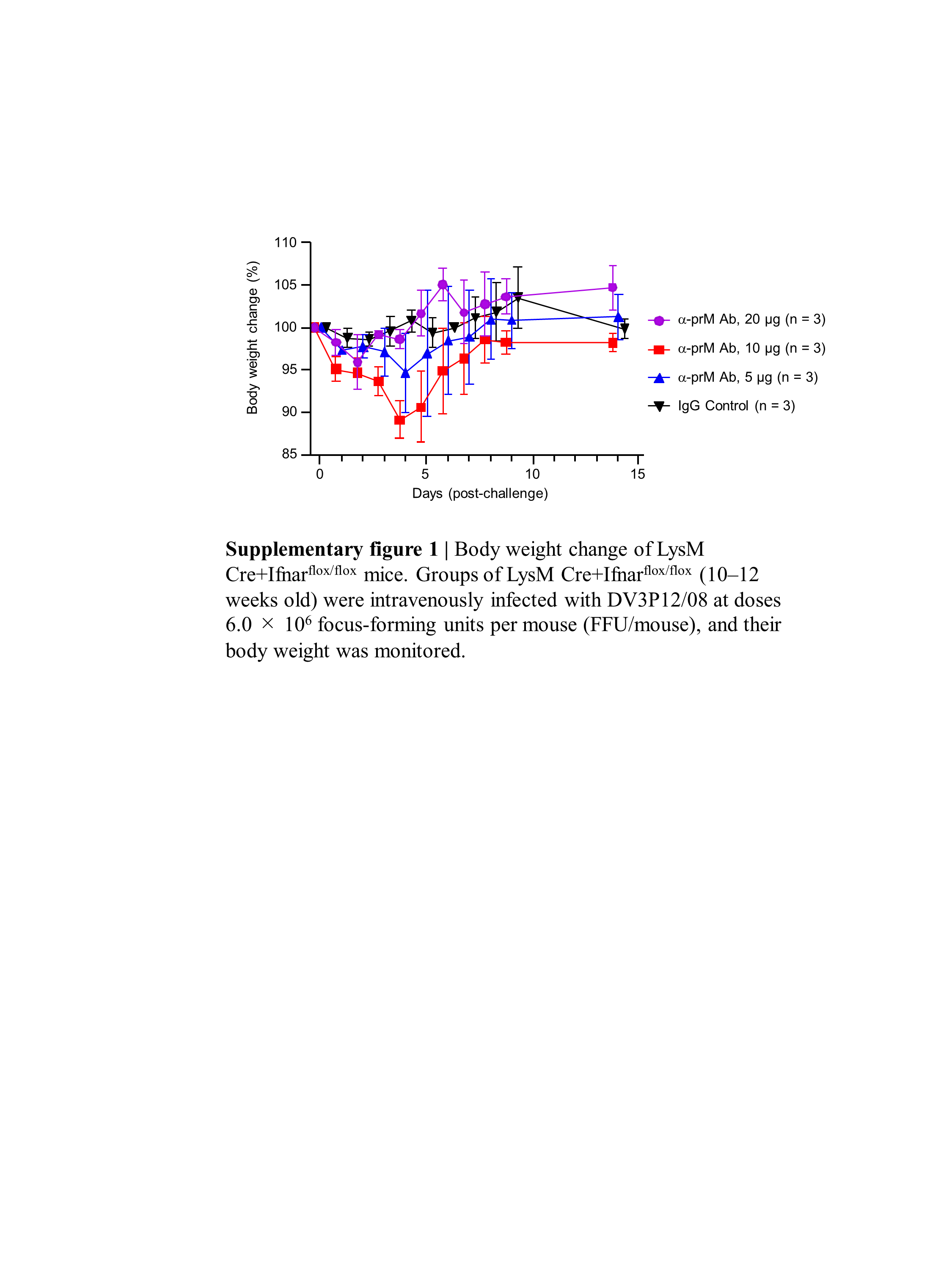

Supplement: Supplementary file 3 [file Image_1.TIF]
